# Supplementary material for: Sensitive and powerful single-cell RNA sequencing using mcSCRB-seq
Source: Nat Commun. 2018 Jul 26;9:2937. doi: 10.1038/s41467-018-05347-6 (PMC6062574; doi:10.1038/s41467-018-05347-6)
Supplement: Supplementary file 1 — Supplementary Information [file 41467_2018_5347_MOESM1_ESM.pdf]

Supplementary Information

Sensitive and powerful single-cell RNA sequencing using mcSCRB-seq

Bagnoli et al.

Supplementary Figure 1

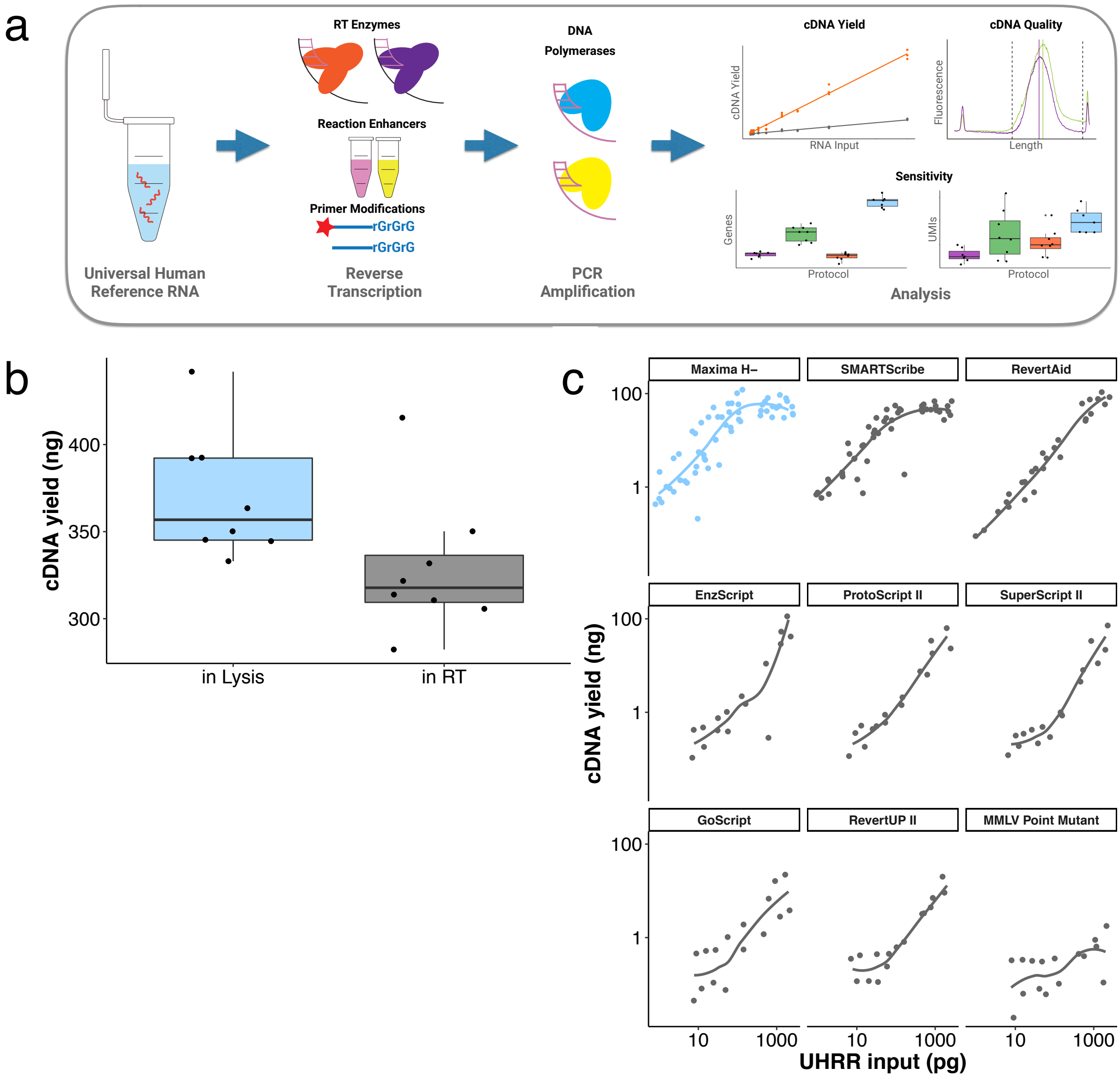

Supplementary Figure 1: Schematic overview and optimization of reverse transcription

a) Low amounts (1-1000pg) of universal human reference RNA (UHRR) were used in optimization experiments. We assessed components affecting reverse transcription and PCR amplification with respect to cDNA yield and cDNA quality and verified effects on gene and transcript sensitivity by sequencing scRNA-seq libraries to develop the mcSCRB-seq protocol.

b) cDNA yield (ng) after reverse transcription with oligo-dT primers already in the lysis buffer ("in Lysis") or separately added before reverse transcription ("in RT"). Each dot represents a replicate and each box represents the median and first and third quartiles. The condition selected for the final mcSCRB-seq protocol is highlighted in blue.

c) cDNA yield (ng) dependent on varying UHRR input using 9 different RT enzymes. Each dot represents a replicate. Lines were fitted using local regression. The condition selected for the final mcSCRB-seq protocol is highlighted in blue.

Supplementary Figure 2

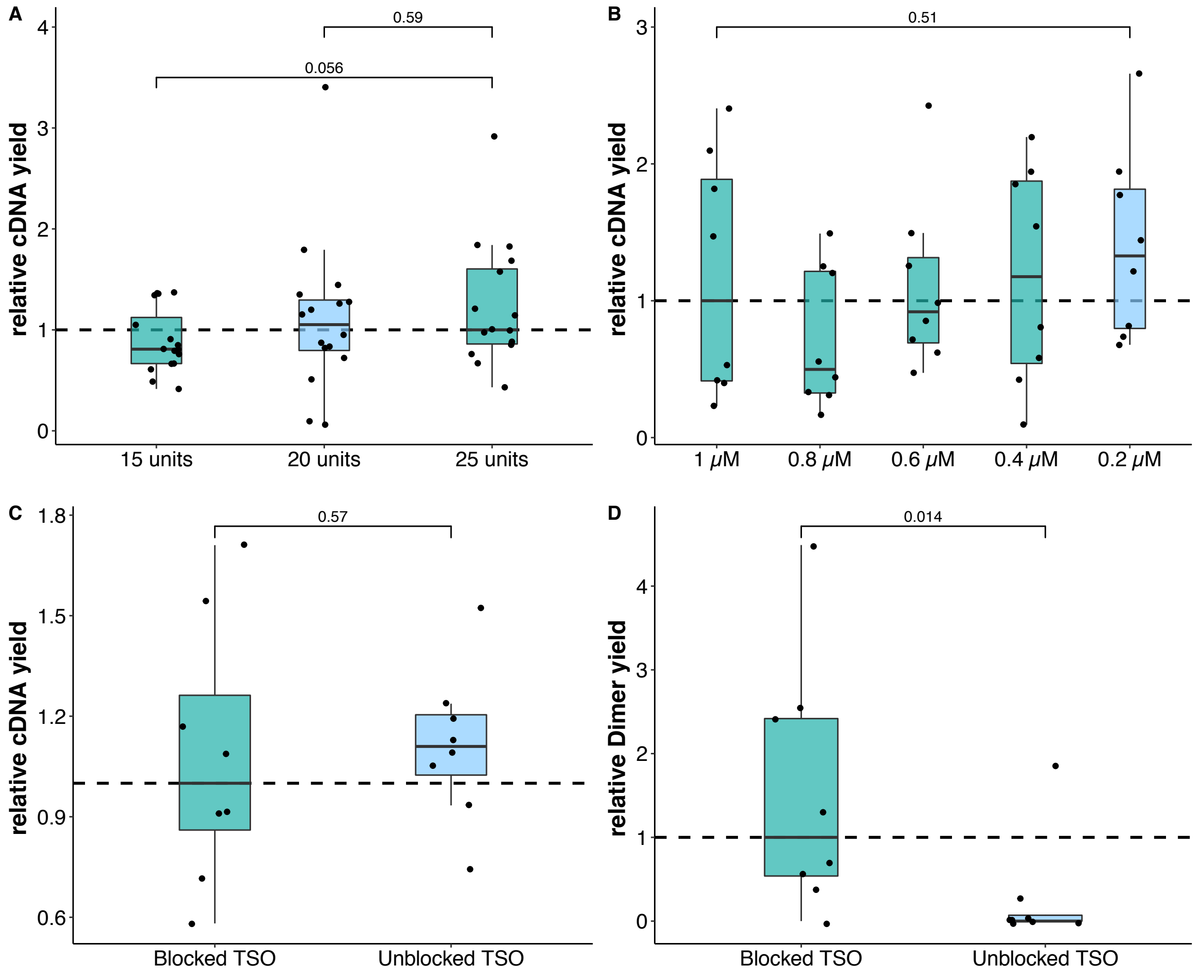

Supplementary Figure 2: Optimization of reverse transcription conditions.
Shown are relative cDNA yields after reverse transcription and PCR amplification of UHRR using:
a) varying amounts of reverse transcriptase enzyme (15-25 units, Maxima H-; 1 ng UHRR input per replicate)
b) varying amounts of oligo-dT primer (E3V6; 1 ng UHRR input per replicate)
c) blocked or unblocked Template switching oligo (TSO, E5V6; 10 pg UHRR per replicate)
d) relative primer dimer yield using blocked or unblocked Template switching oligo (TSO, E5V6) estimated using no-input controls (see Methods).
All values are relative to the median of the condition used in the original SCRB-seq protocol[1], which is indicated by a dashed horizontal line. Each dot represents a replicate and each box represents the median and first and third quartiles method. Numbers above boxes indicate p-values (Welch Two Sample t-test).
Optimized conditions selected for the mcSCRB-seq protocol are marked in blue.

Supplementary Figure 3

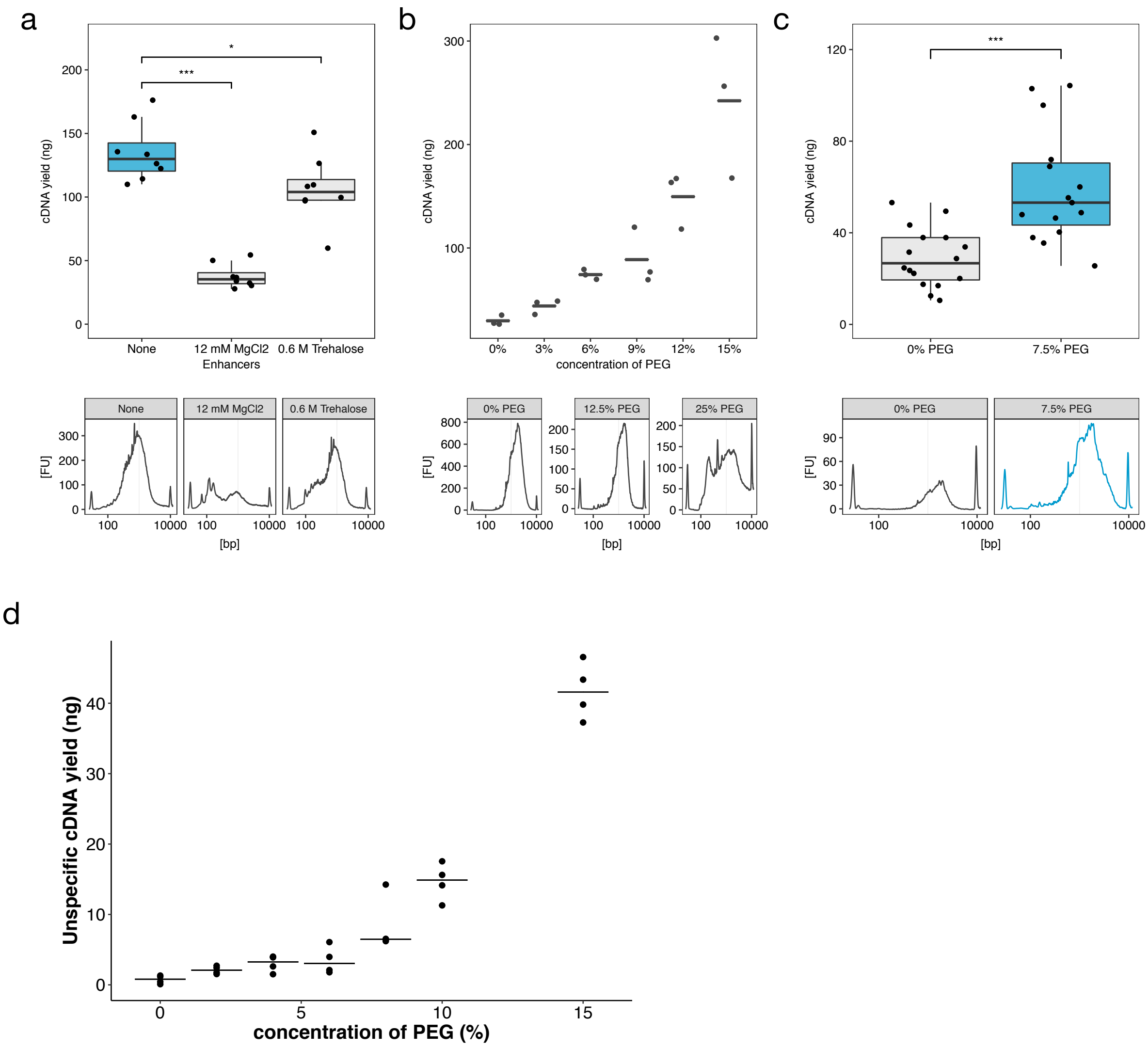

Supplementary Figure 3: Reverse transcription yield is increased by molecular crowding.

cDNA yield as well as representative length distributions (Bioanalyzer traces, bottom) using various additives in the reverse transcription and template switching reaction.

Each dot represents a replicate, lines represent the median and boxes the first and third quartile. Stars above boxes indicate p-values < 0.05 (Welch Two Sample t-test)

a) Influence of MgCl2 and Trehalose on cDNA synthesis (1 ng UHRR input per replicate; 21 PCR cycles).

b) Concentration-dependent influence of PEG 8000 on cDNA yield (100 pg UHRR input per replicate; 23 PCR cycles).

c) Effect of 7.5% PEG 8000 (100 pg UHRR input per replicate; 23 PCR cycles).

d) Concentration-dependent generation of unspecific reverse transcription products (0 pg UHRR input per replicate; 23 PCR cycles).

The conditions selected for the final mcSCRB-seq protocol are highlighted in blue.

Supplementary Figure 4

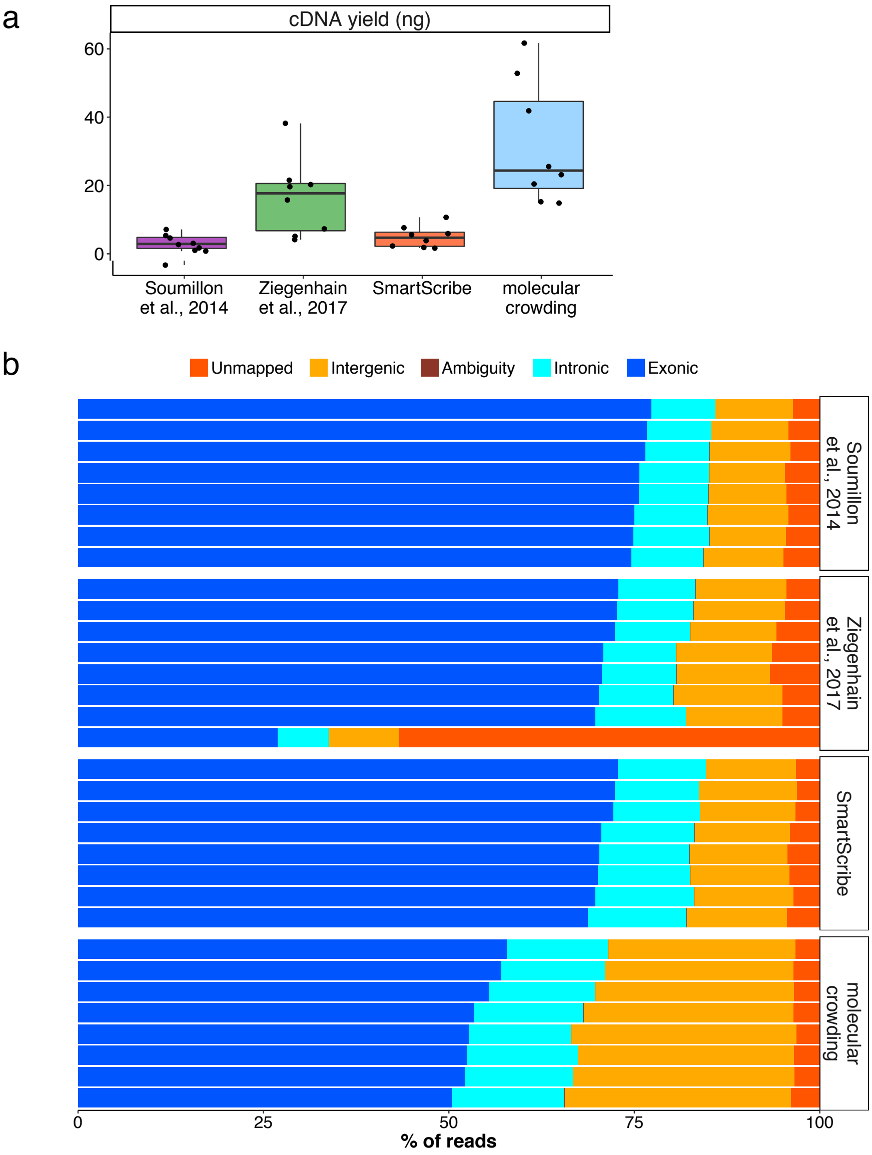

Supplementary Figure 4: Sequencing of UHRR samples.
10 pg of UHRR where used as input for eight replicates for each of the four protocol variants (Supplementary Table 1).
a) cDNA yield (ng) after PCR amplification per method. Each dot represents a replicate and each box represents the median and first and third quartiles per method.
b) Libraries were generated and sequenced from the above cDNA, downsampled to one million reads per library and mapped. Shown are the percentage of sequencing reads that cannot be mapped to the human genome (red), mapped to ambiguous genes (brown), mapped to intergenic regions (orange), inside introns (teal) or inside exons (blue).
Note the higher fraction of reads mapping to intergenic regions, especially in the molecular crowding condition. As UHRR is provided as DNAse-digested RNA, these reads are likely derived from endogenous transcripts, although it is unclear why these are proportionally more detected than annotated transcripts only in the molecular crowding protocol. This is also not generally observed for molecular crowding conditions, as SCRB-seq and mcSCRB-seq protocols have the same fraction (~25%) of intergenic reads mapped when single mouse ES cells are used (Supplementary Figure 7c).

Supplementary Figure 5

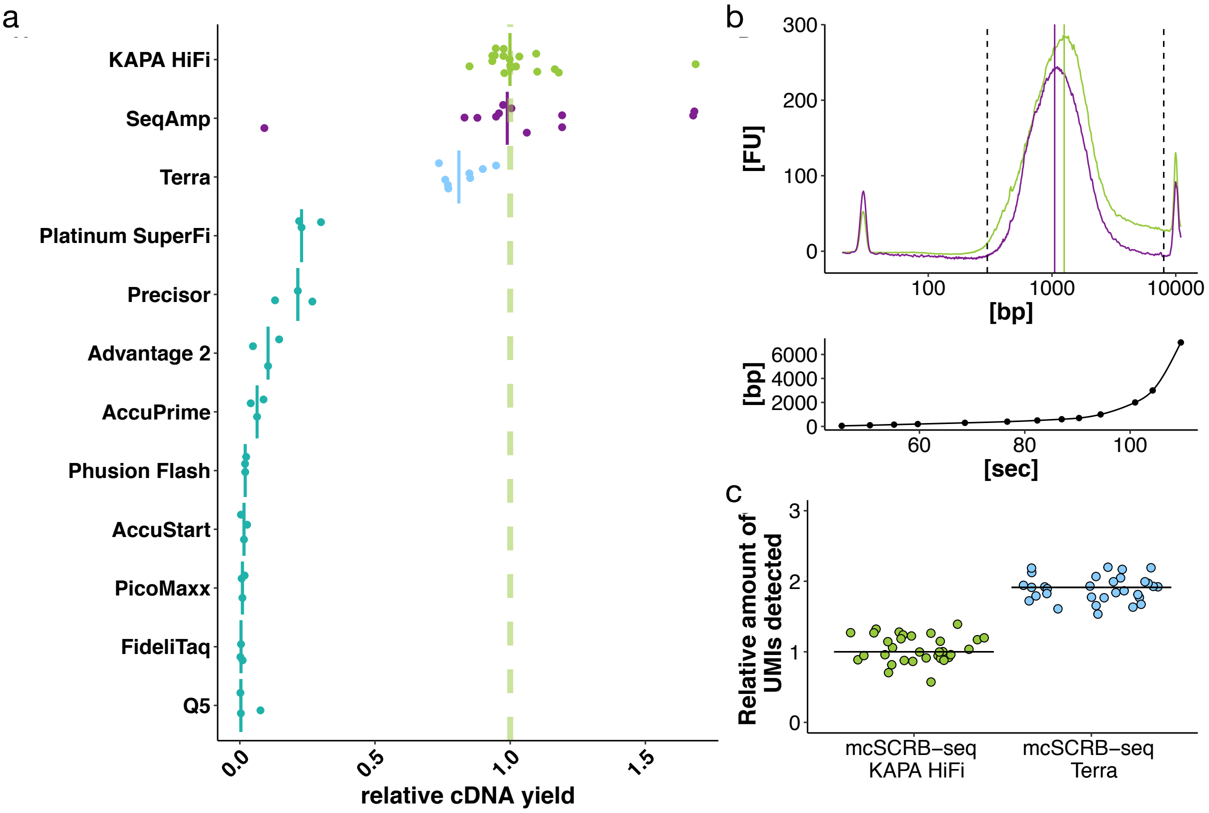

Supplementary Figure 5: Optimization of PCR amplification.

a) Relative cDNA yield after reverse transcription of 1 ng UHRR and amplification using different polymerase enzymes or ready mixes. All values are relative to the median of KAPA HiFi which is indicated by a dashed vertical line, as this was used in the SCRB-seq protocol variant of Ziegenhain et al.[2]. Solid vertical lines indicate the median for each polymerase.

b) Top: Representative length quantification of cDNA libraries amplified with KAPA HiFi (green) or SeqAmp (purple) as quantified by capillary gel electrophoresis (Agilent Bioanalyzer). Solid vertical lines depict the ranked mean length for each library within the region marked with dashed vertical lines. Bottom: Depiction of time length model (spline fit) used to analyze capillary gel electrophoresis via the ladder. Each dot represents a ladder peak with known length (bp) and measurement time (sec).

c) Relative amount of detected UMIs in single mESCs (J1) downsampled to 1 million reads using KAPA-HiFi or Terra for cDNA amplification. For both conditions, molecular crowding conditions (7.5% PEG 8000) were used during reverse transcription. Each dot represents a cell and horizontal lines indicate the median per polymerase.

Supplementary Figure 6

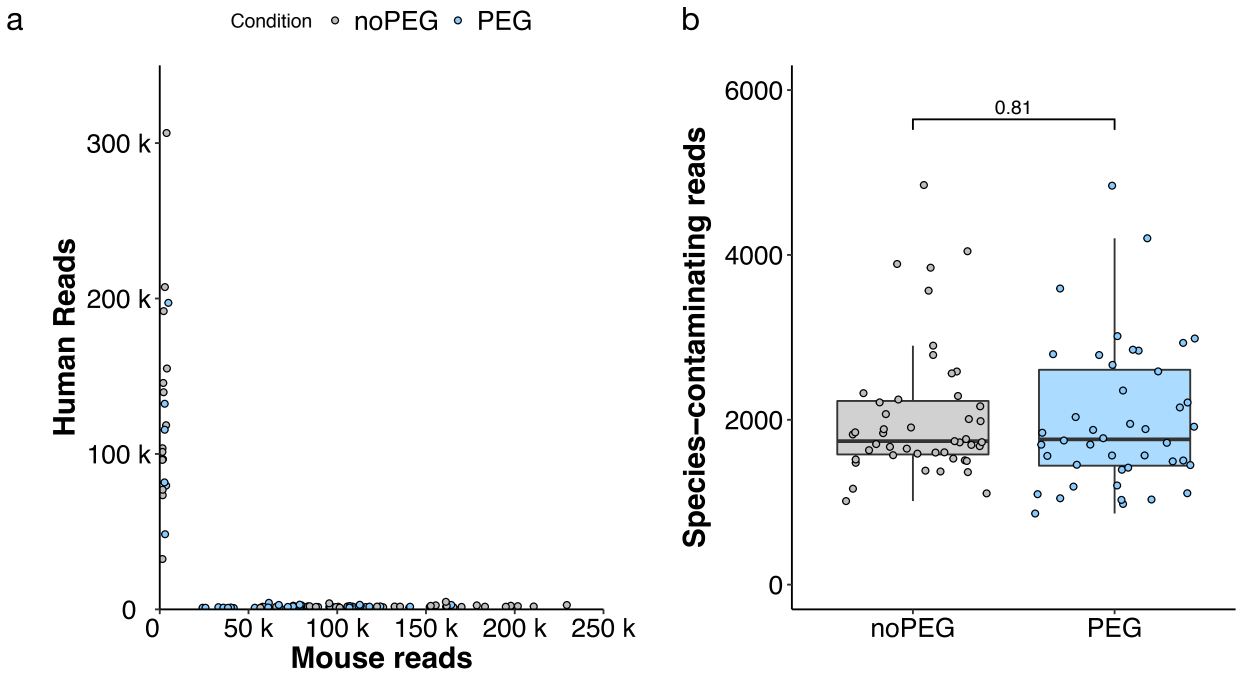

Supplementary Figure 6: Species mixing experiment for mcSCRB-seq
Human induced pluripotent stem cells and Mouse embryonic stem cells were mixed and sorted in a 96-well plate. cDNA was synthesized using the mcSCRB-seq protocol in absence and presence of PEG.
a) For each cell barcode, uniquely aligning reads to human or mouse gene features are shown in a dot plot. No doublets were observed, as expected from single-cell purity FACS sorting.
b) Each cell barcode was classified to be a human or mouse cell. Shown are the number of reads aligning to the wrong species for each of the cell barcodes. There is no significant difference between the protocols with and without PEG (two-sided t-test, p-value=0.81).

Supplementary Figure 7

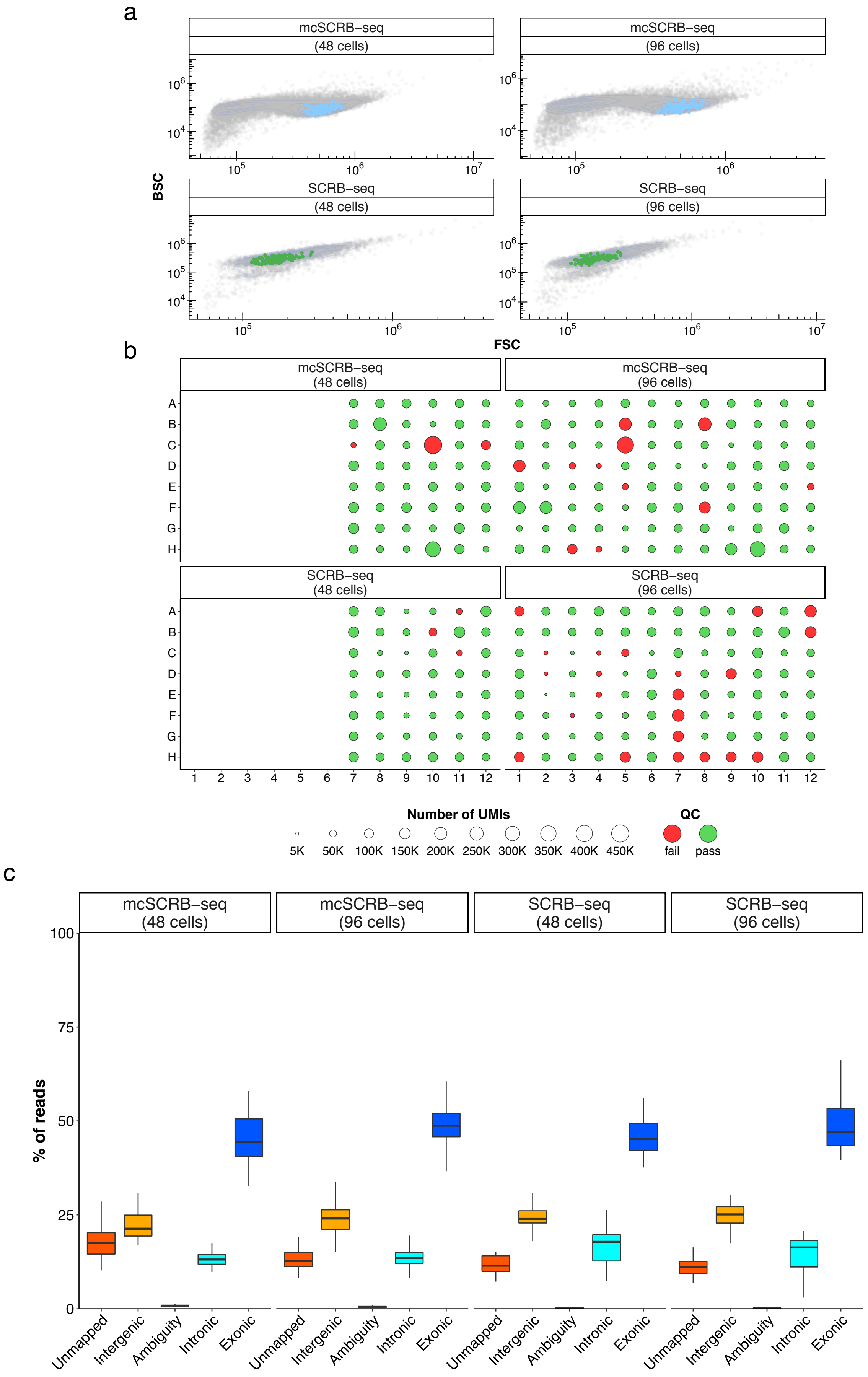

Supplementary Figure 7: Libraries from single mESCs generated with mcSCRB-seq and SCRB-seq protocols.

a) Scatter plots showing FACS data with forward (FS(c) and backward (BS(c) scatter intensities of one vial of mESCs (JM8) resuspended in PBS (mcSCRB-seq) or resuspended in RNAProtect Cell Reagent (SCRB-seq). Each dot represents an event. Coloured dots represent events that were sorted for scRNA-seq libraries in the four plates as depicted in **b**.

b) UMI counts for each cell by method (SCRB-seq/ mcSCRB-seq) and replicate (48 cells/ 96 cells) are shown in their respective position in 96-well plates. Point sizes indicate the number of detected UMIs. Colouring indicates whether a cell passed (green) or failed (red) the Quality Control (QC) as described (see Methods).

c) Percentage of reads that cannot be mapped to the human genome (red), are mapped ambiguously (brown), are mapped to intergenic regions (orange), inside introns (teal) or inside exons (blue). Each box represents the median and first and third quartiles of cells that passed QC for each method.

Supplementary Figure 8

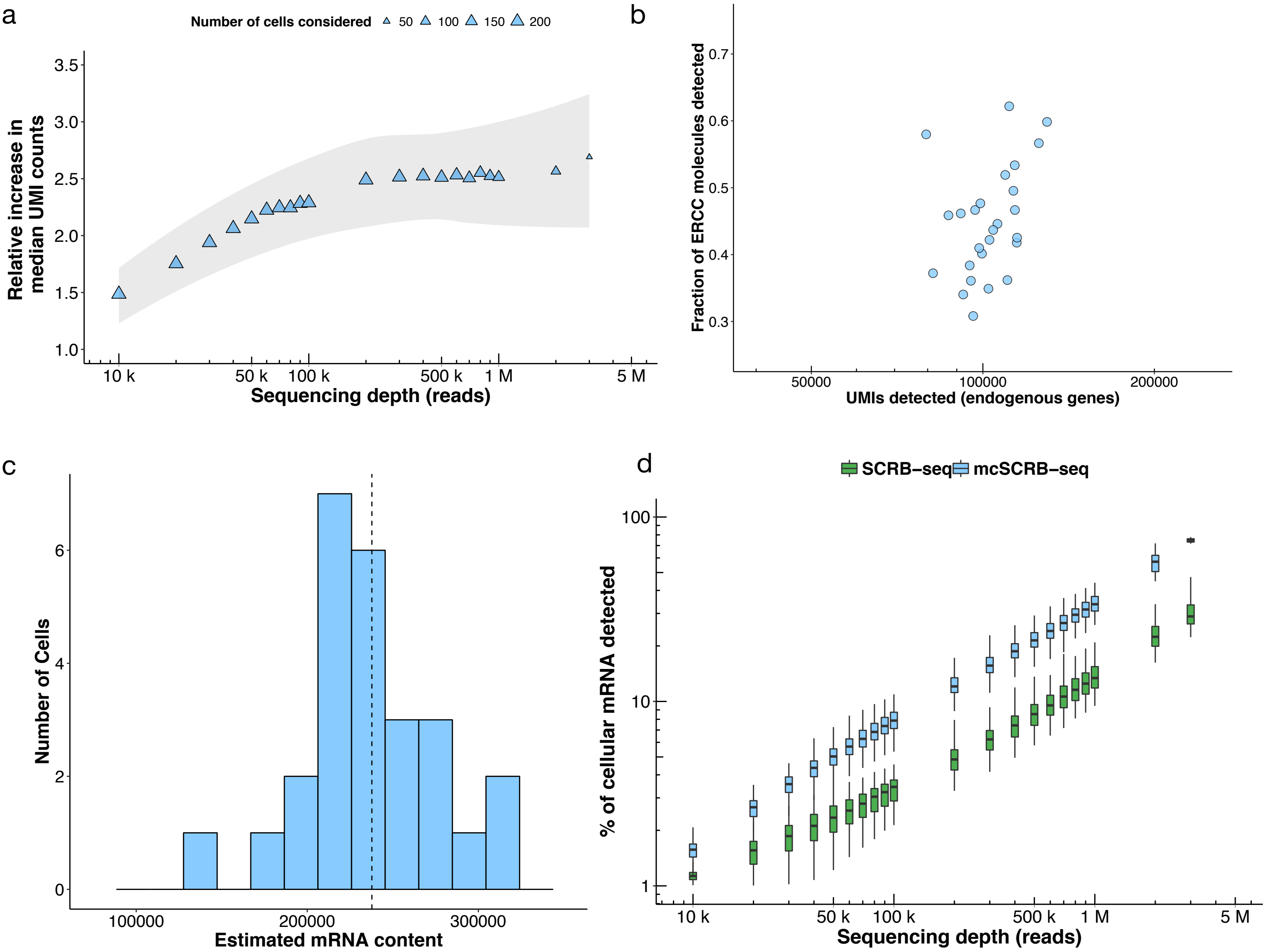

Supplementary Figure 8: Sensitivity of SCRB-seq and mcSCRB-seq protocols.
a) Relative increase in the median of detected UMIs dependent on raw sequencing depth (reads) using mcSCRB-seq compared to SCRB-seq. Each symbol represents the median over all cells at the given sequencing depth. The size of symbols depicts the number of cells (SCRB-seq + mcSCRB-seq) that were considered to calculate the median. The 95% confidence interval of a local regression model is depicted by the shaded area.
b) For each mcSCRB-seq cell that could be downsampled to 2 million reads, the number of UMIs from endogenous genes is plotted on the x axis (median at 102,282 UMIs per cell) and the fraction of UMI- ERCCs from the total amount of spiked-in ERCCs (70,000) is plotted on the y-axis (median 0.49). These values where used to calculate the histogram shown in
c) where for each cell the number of endogenous UMIs is divided by the fraction of ERCCs that were detected in that cell. Using the median of this distribution (dotted line) was set at 100% for the graph in
d) in which the percentage of cellular mRNAs is plotted for each cell at different sequencing depths.

Supplementary Figure 9

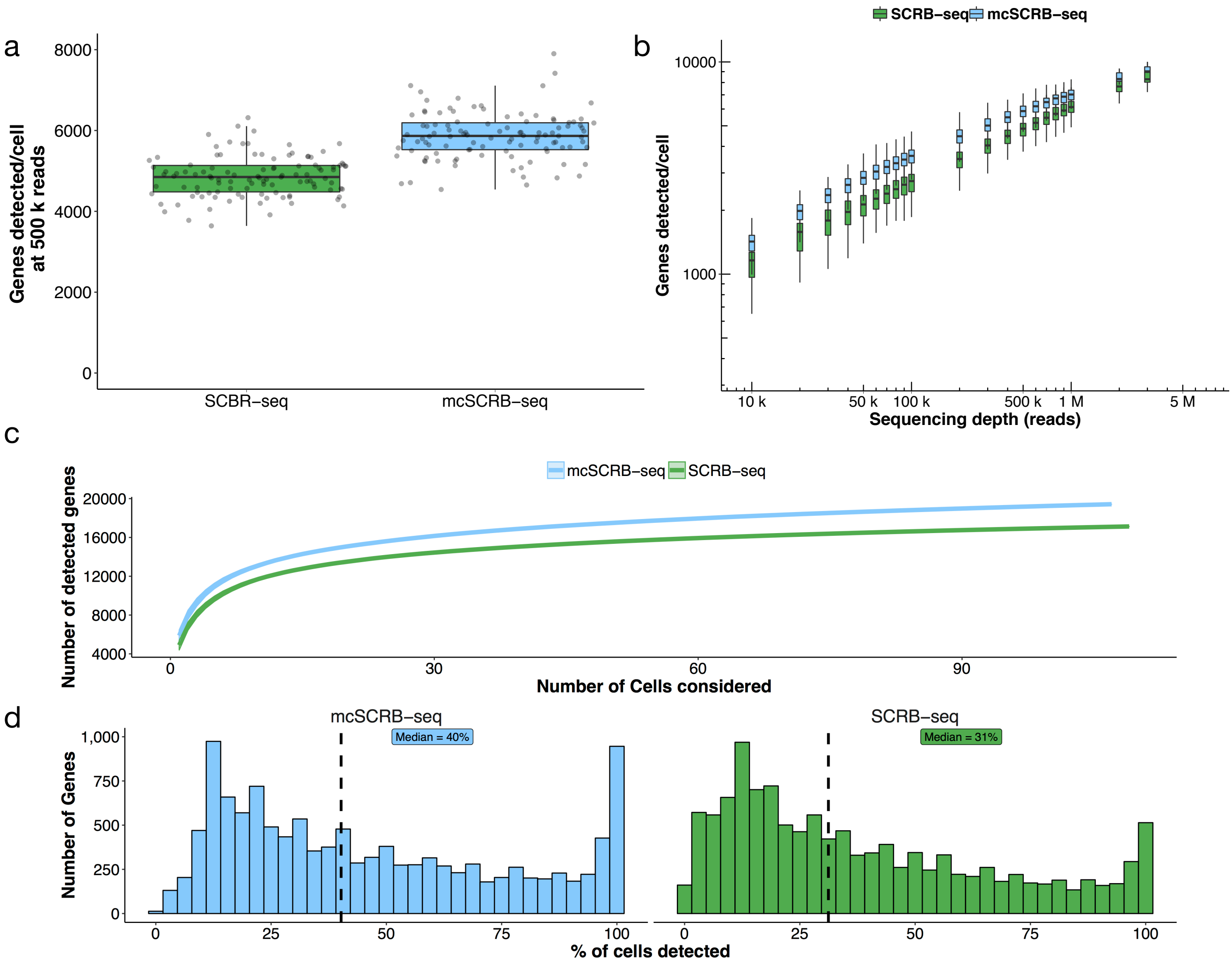

Supplementary Figure 9: Sensitivity of SCRB-seq and mcSCRB-seq protocols by genes.

a) Number of detected genes per cell and method (SCRB-seq/mcSCRB-seq) at a sequencing depth of 500,000 reads per cell (downsampled). Each dot represents a cell and each box represents the median and first and third quartiles.

b) Number of detected genes per cell and method (SCRB-seq/mcSCRB-seq) dependent on sequencing depth (reads). Each box represents the median and first and third quartiles per sequencing depth and method. Sequencing depths and genes are plotted on a logarithmic axis (base 10).

c) Number of detected genes at a sequencing depth of 500,000 reads per cell (downsampled) dependent on the number of cells considered.

d) Gene detection reproducibility is displayed as the fraction of cells detecting a given gene. Dashed line and label indicate the median of the distribution.

Supplementary Figure 10

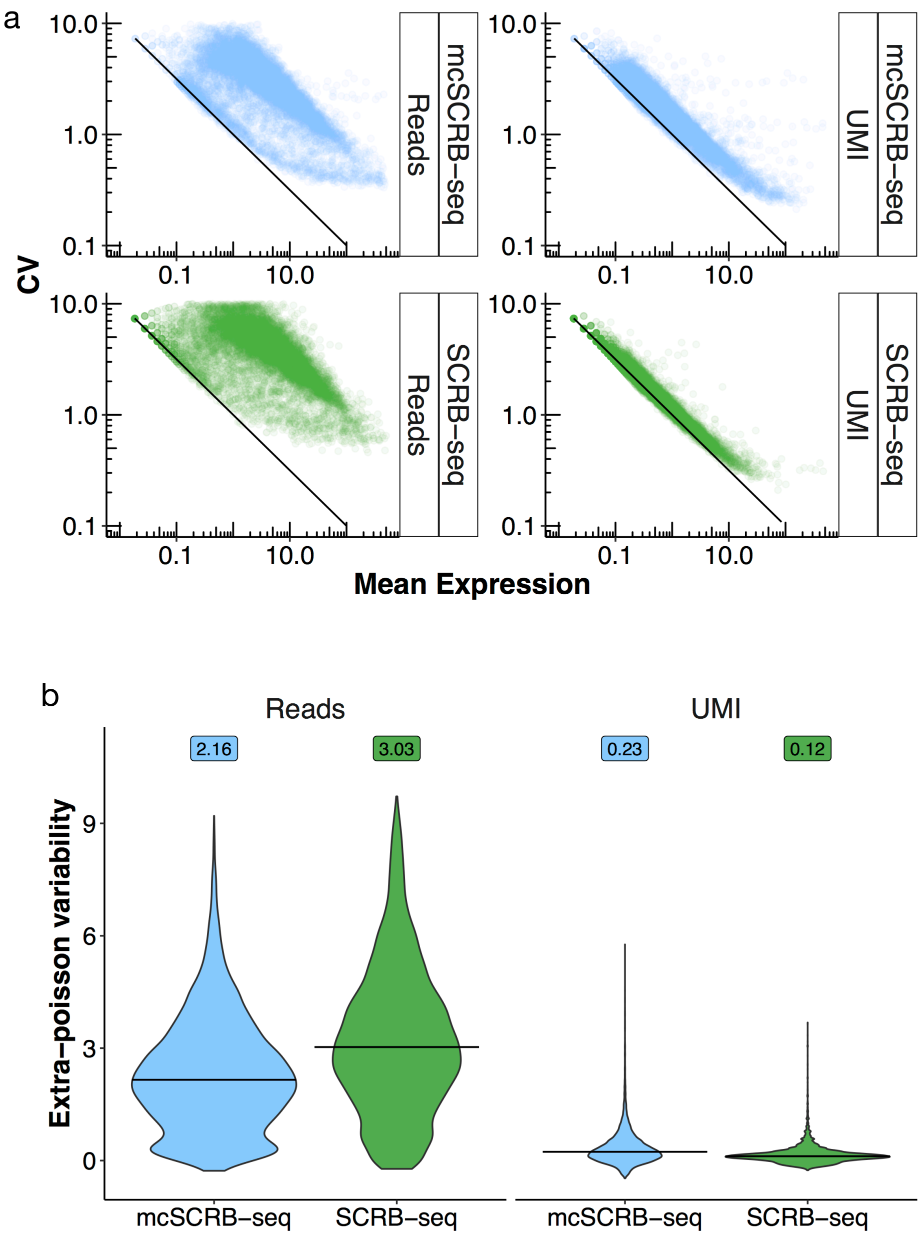

Supplementary Figure 10: Variation parameters of SCRB-seq and mcSCRB-seq protocols by genes.

Variation and mean were calculated for each gene and method in cells downsampled to 500,000 reads using either UMIs per gene or reads per gene.

a) Gene-wise mean and coefficient of variation (standard deviation/mean) from all cells are shown as scatterplots for all methods based on read counts or UMIs. The black line indicates variance according to the poisson distribution.

b) Extra-Poisson variability across 12,086 reliably detected genes (detected in > 10% of cells) was calculated by subtracting the expected amount of variation due to Poisson sampling from the coefficient of variation (CV) measured in read-count or UMI quantification. Distributions are shown as violin plots and medians are shown as bars. Numbers indicate the median for each distribution.

Supplementary Figure 11

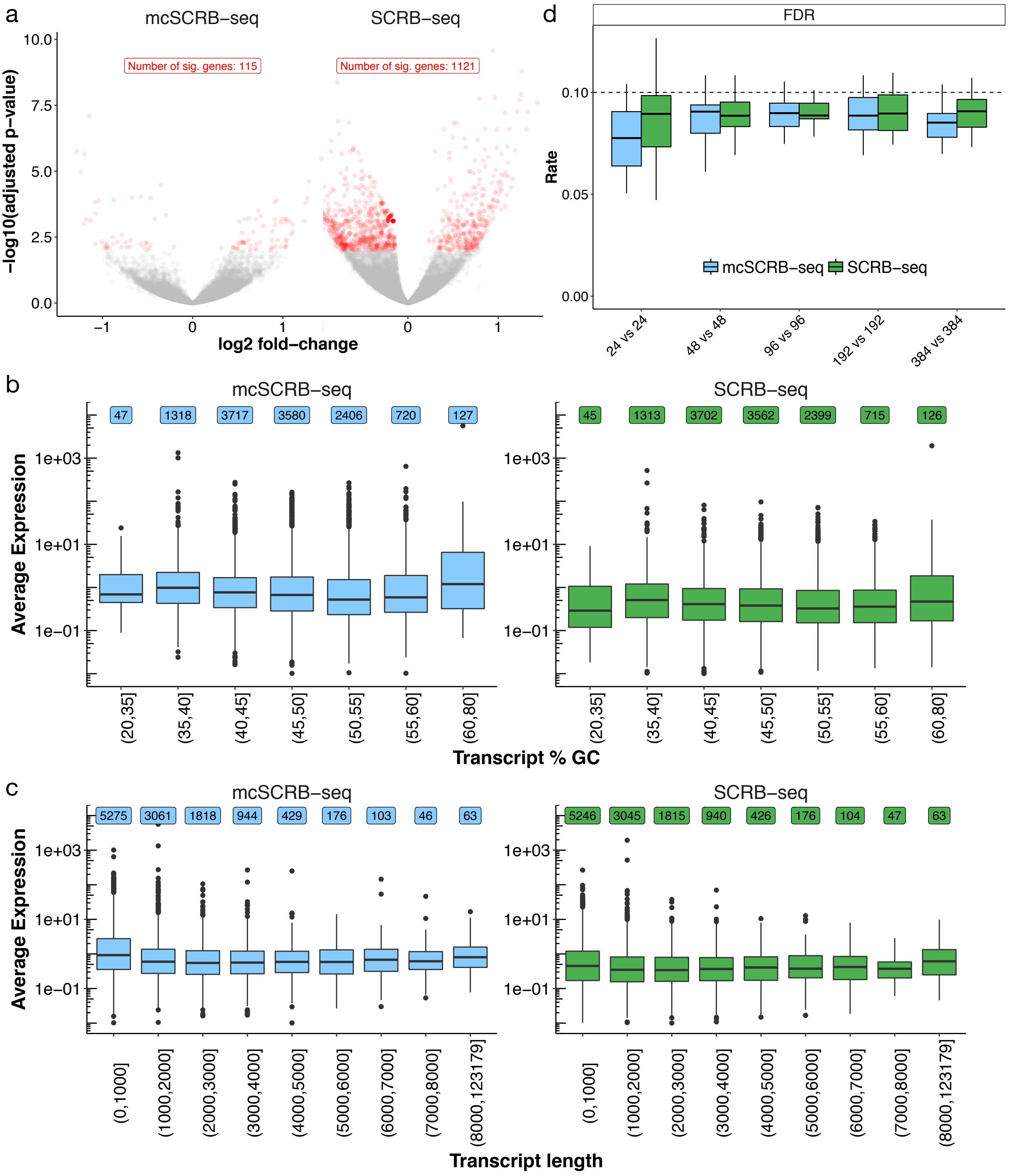

Supplementary Figure 11: Batch effects, biases and power analysis of SCRB-seq and mcSCRB-seq protocols

a) Volcano plots show differentially expressed genes between plates for each method. Points in red depict significantly differentially expressed genes (limma-voom; FDR < 0.01). Red labels show the number of differentially expressed genes between batches.

b) Average detected gene-wise expression levels (log normalized UMI) dependent on GC content of each transcript. Transcripts are grouped in 7 bins of GC content. Each dot represents an outlier and each box represents the median and first and third quartiles.

c) Average detected gene-wise expression levels (log normalized UMI) dependent on transcript length. Transcripts lengths are grouped in 7 bins and number of genes in each bin are indicated. Each dot represents an outlier and each box represents the median and first and third quartiles.

d) Power simulations were performed using the powsimR package[3] from empirical parameters estimated at 500,000 raw reads per cell. For SCRB-seq and mcSCRB-seq, we simulated n-cell two-group differential gene expression experiments with 10% differentially expressed genes. Shown is the false discovery rate ("FDR") for sample sizes
n = 24, n = 48, n = 96, n = 192 and n = 384 per group. The corresponding true positive rate is shown in Figure 2b. Boxplots represent the median and first and third quartiles of 25 simulations. Dashed lines indicate the desired nominal level.

Supplementary Figure 12

a

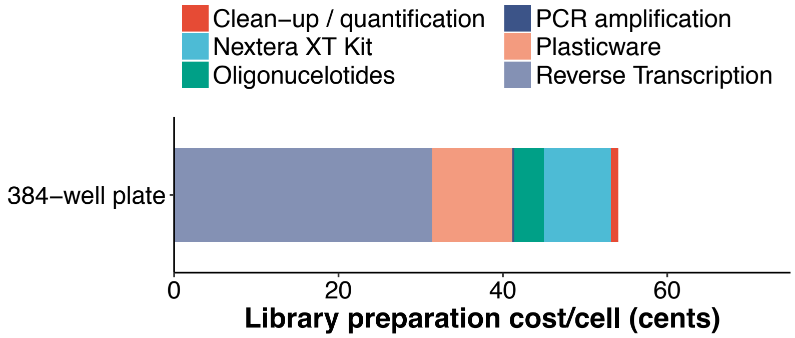

b

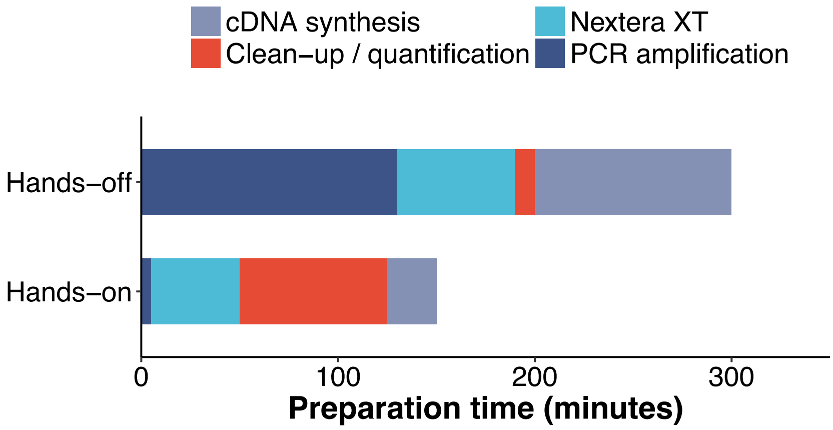

Supplementary Figure 12: Costs and preparation time of mcSCRB-seq
a) Library preparation costs (Eurocents) per cell. Colors indicate the consumable type based on list prices (see Supplementary Table 3). Costs also apply if four 96-well plates are pooled for PCR amplification and Nextera
b) Library preparation time for one 96-well plate of mcSCRB-seq libraries was measured for bench times (“Hands-on”) and incubation times (“Hands-off”). Colors indicate the library preparation step. The total time was 7.5 hours. (see Supplementary Table 4)

Supplementary Figure 13

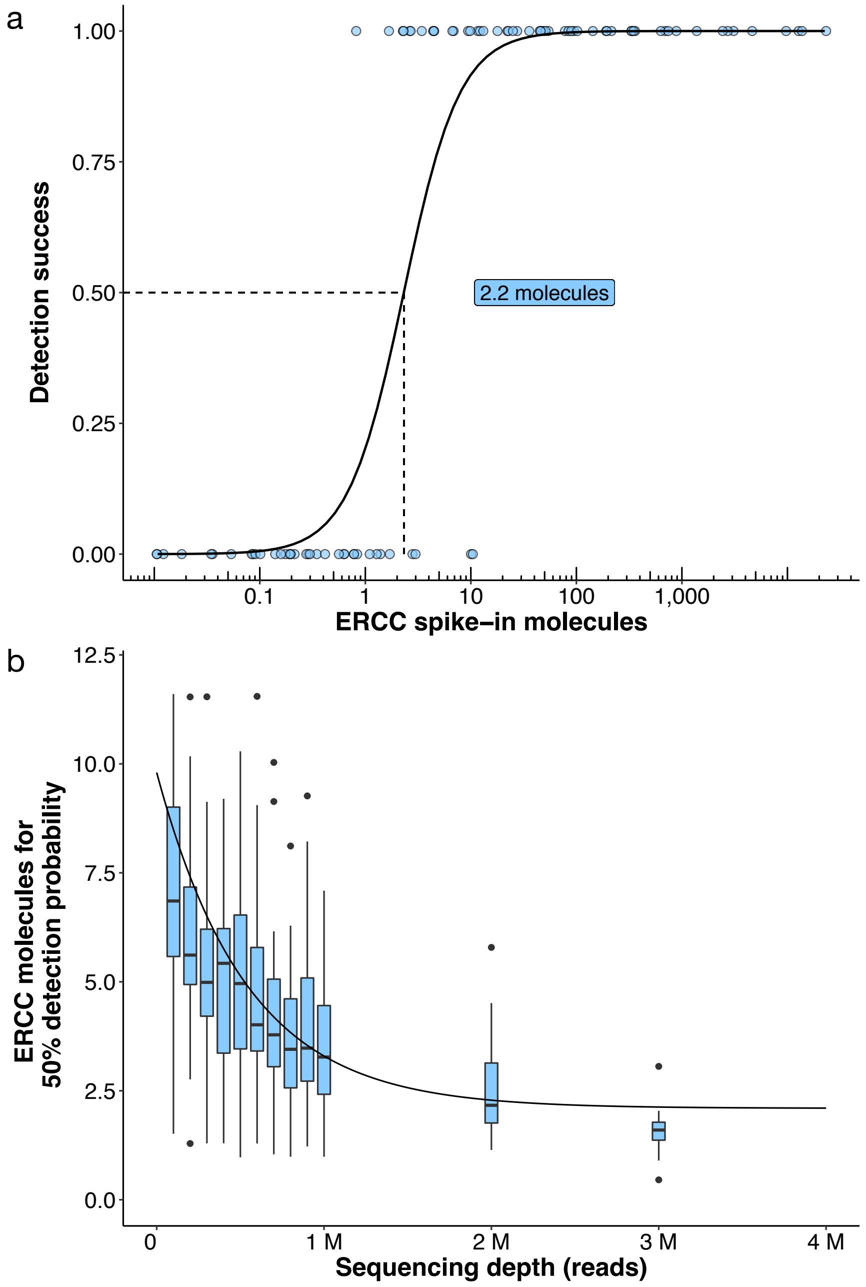

Supplementary Figure 13 : Comparison of mcSCRB-seq to other scRNA-seq data based on ERRCC spike-in detection probability

a) Shown is the detection (0 or 1) of the 92 ERCC transcripts in an average cell processed with mcSCRB-seq at 2 million reads coverage. Points and solid line represent the ERCC genes with their logistic regression model. Dashed lines and label indicate the number of ERCC molecules required for a detection probability of 50%.

b) Number of ERCC molecules required for 50% detection probability dependent on the sequencing depth (reads) for mcSCRB-seq. Each each box represents the median, first and third quartiles of cells per sequencing depth with dots marking outliers. A non-linear asymptotic fit is depicted as a solid black line.

Supplementary Figure 14

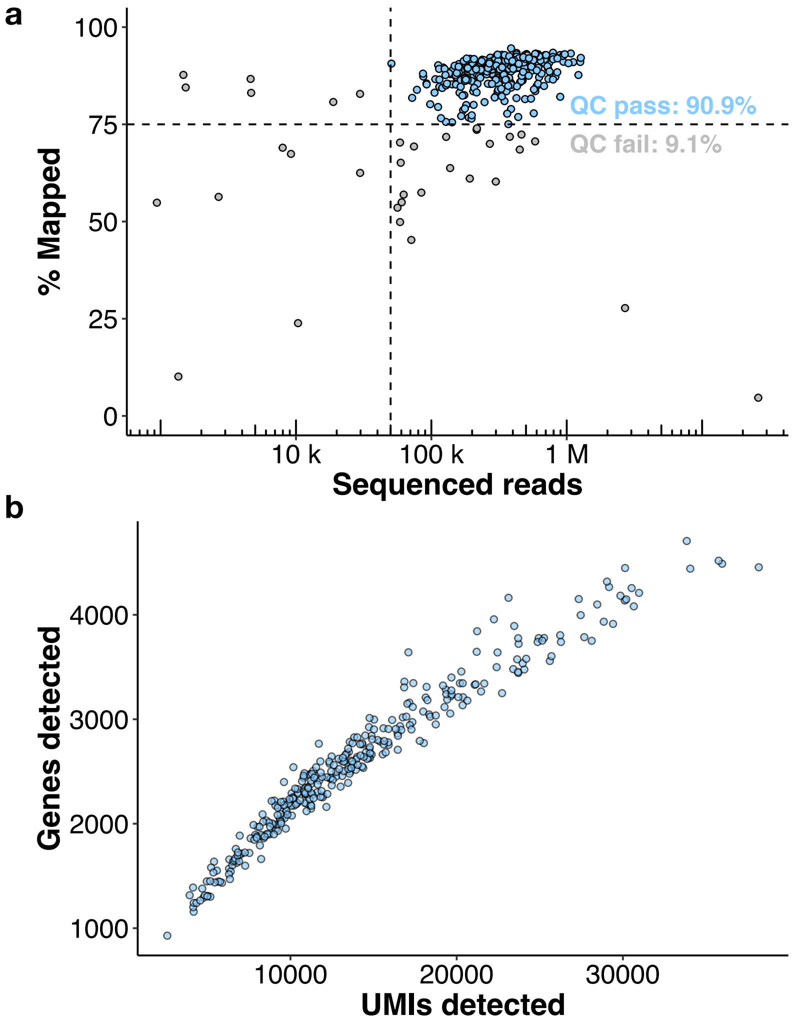

Supplementary Figure 14: Quality control of PBMC data

a) Scatter plot shows each of the 384 sequenced PBMC cells with the number of sequenced reads and the % of those reads mapped to the human genome. Dashed lines indicate quality filtering cut-offs chosen. Colors indicate QC passed cells (blue) or discarded cells (grey).

b) Cell-wise detected genes (>=1 UMI) and detected UMIs are shown for all cells that passed quality control (n=349).

Supplementary Table 1

protocol variant	Soumillon	Ziegenhain	SmartScribe	molecular crowding
Reverse transcriptase	Maxima H-	Maxima H-	SmartScribe	Maxima H-
Buffer enhancer	none	none	none	7.5% PEG
PCR polymerase	Advantage2	KAPA HiFi	KAPA HiFi	KAPA HiFi

Supplementary Table 1: Overview of used enzymes and enhancers in UHRR based experiments.

Supplementary Table 2

	**SCRB-seq**	**mcSCRB-seq**
Lysis	Phusion HF	Phusion HF + Proteinase K + oligo-dT primers
Cell suspension	RNAprotect	PBS
Proteinase K	Ambion	Clontech
oligo-dT concentration	1 µM	0.2 µM
reverse transcription volume	2 µl	10 µl
RT amount	25 U	20 U
RT enhancer	none	7.5% PEG
TSO modification	5'-blocking	none
TSO concentration	1 µM	2 µM
Pooling	Zymo Clean & Concentrator	magnetic beads
PCR polymerase	KAPA HiFi	Terra direct
PCR cycles	18-21	13-15
Protocol speed	2 days	1 day
Cost per cell	1-2 €	0.4-0.6 €

Supplementary Table 2: Overview of the key differences between SCRB-seq as used in Ziegenhain et al.[2] and mcSCRB-seq (this work).

Supplementary Table 3

consumable	price/unit	# 384 plates	price/384 plate
Barcode oligo-dT	24.000,00 €	5000	4,80 €
TSO E5V6unblocked	453,40 €	50	9,07 €
Maxima RT	554,00 €	5	110,80 €
Exonuclease I	327,00 €	1000	0,33 €
Clontech Terra	551,00 €	800	0,69 €
Nextera XT	3.002,00 €	96	31,27 €
dNTPs	1.236,00 €	125	9,89 €
Beads	20,00 €	10	2,00 €
Picogreen	542,00 €	400	1,36 €
PCR Seal	500,00 €	1000	0,50 €
PCR Plate/96	140,00 €	0	0,00 €
PCR Plate/384	195,00 €	25	7,80 €
Tips/96	36,50 €	0	0,00 €
Robotic tips/384	290,00 €	10	29,00 €
Total			207,50 €
**Total/cell**			**0,54 €**

Supplementary Table 3. Detailed overview of costs for mcSCRB-seq.

Supplementary Table 4

**Task**	**Hands-on (min)**	**Hands-off (min)**	**suggested start time**	**Stopping point?**	**Note**
Prepare workplace	10		09:00		
Proteinase K digest	10	10	09:10		Meanwhile prepare RT Master-Mix
Dispense RT Mix	5		09:30		
RT		90	09:35		
Pool + Clean-up	35	10	11:05	<72h @ 4°C	
ExoI		30	11:50		
PCR set-up	5,00		12:20		
PCR		100	12:25		
PCR clean-up	20,00		14:05	1 week @ 4°C or long-term @ -20 °C	
Quantify cDNA	5,00		14:25		
Nextera: Transposition + PCR set-up	20	10	14:30		
Nextera XT PCR		40	15:00		
PCR clean-up	15,00		15:40	1 week @ 4 °C or long-term @ -20 °C	
Gel-excision & clean-up	25	10	15:55	1 week @ 4 °C or long-term @ -20 °C	
			16:30		
**total time**	**150**	**300**			

Supplementary Table 4. Detailed overview of hands-on and hands-off time necessary to create a sequenceable mcSCRB-seq library from one single cell plate.

Supplementary References

1. Soumillon, M., Cacchiarelli, D., Semrau, S., van Oudenaarden, A. & Mikkelsen, T. S. Characterization of directed differentiation by high-throughput single-cell RNA-Seq. *bioRxiv* (2014). doi:10.1101/003236

2. Ziegenhain, C. *et al.* Comparative Analysis of Single-Cell RNA Sequencing Methods. *Mol. Cell* 65**,** 631–643.e4 (2017)

3. Vieth, B., Ziegenhain, C., Parekh, S., Enard, W. & Hellmann, I. powsimR: Power analysis for bulk and single cell RNA-seq experiments. *Bioinformatics* (2017). doi:10.1093/bioinformatics/btx435
